# Virtual Art Therapy: Application of Michelangelo Effect to Neurorehabilitation of Patients with Stroke

**DOI:** 10.3390/jcm12072590

**Published:** 2023-03-29

**Authors:** Roberto De Giorgi, Antonio Fortini, Federica Aghilarre, Federico Gentili, Giovanni Morone, Gabriella Antonucci, Mario Vetrano, Gaetano Tieri, Marco Iosa

**Affiliations:** 1 Casa di Cura Nomentana Hospital, 00013 Rome, Italy; 2 Department of Life, Health and Environmental Sciences, University of L’Aquila, 67100 L’Aquila, Italy; 3 Department of Psychology, University Sapienza of Rome, 00185 Rome, Italy; 4 IRCCS Santa Lucia Foundation, 00179 Rome, Italy; 5 Physical Medicine and Rehabilitation Unit, Sant’Andrea Hospital, Sapienza University of Rome, 00189 Rome, Italy; 6 Virtual Reality Lab, Department of Law and Digital Society, UnitelmaSapienza University, 00161 Rome, Italy

**Keywords:** art therapy, neuroaesthetics, aesthetics, stroke, rehabilitation, Michelangelo effect, immersive virtual reality

## Abstract

In neurorehabilitation, some studies reported the effective use of art therapy for reducing psychological disorders and for enhancing physical functions and cognitive abilities. Neuroaesthetical studies showed that seeing an art masterpiece can spontaneously elicit a widespread brain arousal, also involving motor networks. To combine contemplative and performative benefits of art therapy protocols, we have developed an immersive virtual reality system, giving subjects the illusion that they are able to paint a copy of famous artistic paintings. We previously observed that during this virtual task, subjects perceived less fatigue and performed more accurate movements than when they were asked to color the virtual canvas. We named this upshot the Michelangelo effect. The aim of this study was to test the rehabilitative efficacy of our system. Ten patients with stroke in the subacute phase were enrolled and trained for one month with virtual art therapy (VAT) and physiotherapy. Their data were compared with those of ten patients matched for pathology, age and clinical parameters, trained only with conventional therapy for the same amount of time. The VAT group showed a significantly higher improvements in the Barthel Index score, a measure of independency in activities of daily living (66 ± 33% vs. 31 ± 28%, *p* = 0.021), and in pinching strength (66 ± 39% vs. 18 ± 33%, *p* = 0.008), with respect to the group treated with conventional rehabilitation.

## 1. Introduction

In 2019, the World Health Organization (WHO) published a review of more than 3000 studies stating that art therapy has a potential value for promoting good health, ameliorating or preventing of a range of mental and physical health conditions, and treating or managing acute and chronic conditions arising across one’s lifespan [1].

Art therapy is also used in neurorehabilitation [2,3], with two main possible approaches: contemplation and production [1]. Contemplation refers, for example, to the observation of visual artworks (such as paintings and sculptures), or to the listening of musical pieces, or their digital reproductions [1]. On the other hand, production refers to a more active process, in which the subject is asked to paint, to sing, or to play an instrument [1]. The latter approach has the advantage of a more active involvement of the patient, but the disadvantage that the product is rarely definable as art (because a common person, affected or not by a disease, is usually not an artist).

Surprisingly, the wide review of WHO [1] about art-therapy did not mention the field of neuroaesthetics and its principles. The discussion was focused on the efficacy of art-therapy, without specific analysis of the neural effects of art on the brain. Neuroaesthetics is a relatively new subfield of the neurosciences, aiming to understand the brain mechanisms engaged during aesthetic and artistic experiences in a wide sense [4]. Most of the studies regarding neuroaesthetics have been conducted in order to investigate as to how the healthy brain responds to artistic stimuli. A widespread brain arousal has been observed, even involving the motor and premotor networks [5,6,7,8]. The reasons for which the simple contemplation of an artistic masterpiece activates the motor areas were found to derive from the possible recognition of emotions displayed by the expressions of painted persons [6], from neuron networks activated by their actions [7], from the ideal possibility to move throughout the represented scene [5], and even from the observer’s empathetic engagement, with a simulation of the motor program that corresponds to the gestures done by the artist to produce the artwork [7,8]. Furthermore, art observation reduces stress and blood pressure, improves cognitive and emotional processes, and distracts from negative experiences [9].

All these findings provide a solid neuroscientific support for the use of art in rehabilitation. and define some principles helpful for defining art therapy protocols. According to these premises, a combined approach of contemplative and performative art therapy could be helpful for neuromotor rehabilitation of people with stroke. To overcome the problem of combining the processes of the contemplation of real artworks and active production, we capitalized on the power of immersive virtual reality (VR) for developing a novel technological system, according to the above-described neuroaesthetic principles. This choice was also motivated by the increasing evidence that highlights the potential and effectiveness of immersive VR in clinical applications [10]. Indeed, immersive VR technology offers many advantages, ranging from its technological aspects to its impact on the human brain and behaviour (see [10] for a recent review). Regarding the first aspect, VR offers a high level of ecological validity and usability, it is easy to use in laboratory and clinical settings, and it is cost-effective [10].

On the other hand, studies conducted in different scientific fields, including psychology, neuroscience, and computer science, largely demonstrated that VR has as a strong influence on human perceptions and cognition. In this line, the sense of presence, i.e., the illusion to feel physically immersed in a virtual environment, which allows for responding in realistic way to the virtual stimuli, represents one of the most important aspects [11,12]. Importantly, the sense of presence can be exploited in terms of boosting the impact of rehabilitation, by inducing sensations in the patients that give an illusion of being in the real world, which can be designed to elicit specific behaviors and exploit specific abilities [13].

Another important aspect is that VR allows a naturalistic interaction between humans and the virtual environment. Indeed, by using the VR headset’s controller, the participants can use their real hands for interacting with the virtual objects represented in the digital environment. In this way, we can easily measure the kinematics of the upper limbs while participants perform the motor tasks. Finally, VR also allows one to simulate many different conditions that are difficult or impossible to reproduce in real life [14], such as giving one the illusion that they are able to paint some of the most famous masterpieces of the history of art [15].

In this light, we previously used VR for implementing and testing a novel task of art therapy for upper limb neurorehabilitation, where healthy subjects and patients with stroke could experience painting, as an illusion, an artistic masterpiece, i.e., the Venus of Botticelli, the Creation of Adam of Michelangelo, and many other famous paintings. We found that subjects made less kinematic errors (related to a trajectory of the hand away from the canvas) and were reported to perceive less fatigue when they unveiled a beautiful artistic painting than when they were simply asked to color the canvas [15]. We called this effect of art the “Michelangelo effect” [15], in analogy with the famous “Mozart effect” of music on cognitive performances [16]. Our previous feasibility study about the VR system also included a pilot trial with four patients, and it showed promising results regarding the therapeutic use of this virtual art therapy system in neurorehabilitation [15].

In the present study, we aimed to test the efficacy of the virtual art therapy (VAT) protocol, based on neuroaesthetic principles and the Michelangelo effect, in order to improve the upper limb recovery in patients with stroke using a pilot protocol, including 12 sessions (3 per week), with respect to conventional physiotherapy.

Based on previous evidence, we expected to find an improvement in the level of upper limb functioning, as well as increased independency regarding the abilities of daily living, in patients who performed the VR protocol, with respect to the group who underwent the conventional physiotherapy.

## 2. Materials and Methods

### 2.1. Participants

Patients with stroke in the subacute phase were enrolled in this study. According to the stroke recovery and rehabilitation roundtable, the early subacute phase is defined as the period between 7 days and 3 months after the stroke event [17]. This timing was in line with the guidelines, as defined by the Italian Ministry: the median time between the stroke event and the beginning of rehabilitation in Italy is 20 days (interquartile range: 13–32 days) [18], according to previous findings reporting that beginning rehabilitation within the first 20 days is associated with a significantly high probability of a better therapeutic response [19]. Inclusion criteria were the following: single ischemic or hemorrhagic stroke in the subacute phase, as confirmed by brain imaging (magnetic resonance imaging or computerized tomography); an ability to understand and follow the instructions given by therapists (Mini Mental State Evaluation ≥24) [20]; manual muscle test total score >4. Exclusion criteria were the following: presence of visual deficits, presence of unilateral spatial neglect, presence of severe comorbidities, and a diagnosed risk of epilepsy. Obviously, even if it was not an inclusion criterion of our study, it should be considered that only patients with stable clinical conditions and who were able to begin the rehabilitation were discharged from acute stroke units and admitted to neurorehabilitation wards [18].

This study was conducted in accordance with the Declaration of Helsinki, and approved by the Institutional Review Board. Signed informed consent was obtained from all subjects involved in the study.

According to the preliminary results obtained in a previous pilot study [15], using the root mean square error as outcome variable, setting the alpha level at 5% and the power at 99%, we found the need to enroll 19 patients in order to obtain statistically significant differences with the Mann–Whitney u-test. So, 10 patients have been enrolled in the experimental group and, another ten patients, matched for age, gender, type of stroke and other clinical parameters, formed the control group.

### 2.2. Study Protocol and Assessment

After the enrollment, patients were assigned to the virtual art therapy group (VATG) or control group (CG). Patients received the same amount of therapy independently by their allocation group. The control group followed the conventional pathway of rehabilitation therapy: 3 sessions per day, each one comprising 1 h, 6 days a week, for one month. According to the needs of the patient, the total amount of 3 h of daily therapy could include not only physical therapy for the upper and lower limbs, as well as for posture and balance, but also cognitive therapy, occupational therapy, speech therapy, and specific therapy for swallowing, bowel, and bladder dysfunctions.

The VATG substituted 12 sessions (3 per week) of upper limb physical therapy, aiming at arm functional recovery, with 12 sessions of virtual art therapy (as described in the next section). The other daily therapies for VATG remained similar to those of CG. All patients were assessed at baseline (after enrollment) and 1 month later (at the end of this period of therapy). The assessment was based on three clinical scales: the Barthel Index (BI), to assess the independency of patients in their activities of daily living (the BI score goes from 0, total dependency, to 100, total independency), the Manual Muscle Test, to assess the strength of the upper limbs (MMT; a score going from 0 to 5 was assigned, regarding the strength of shoulder abduction, elbow flexion and pinching, with a total score going from 0, no force, to 15, normal force), and the Ashworth spasticity scale (with the score going from 0, no spasticity, to 5 in which spasticity does not allow any passive mobilization). The participation in each VAT session was assessed by using the Italian version [21] of the Pittsburgh Rehabilitation Participation Scale [22], with the perceived fatigue additionally assessed by using a numeric rating scale [15].

### 2.3. Virtual Art Therapy

**Figure 1:**
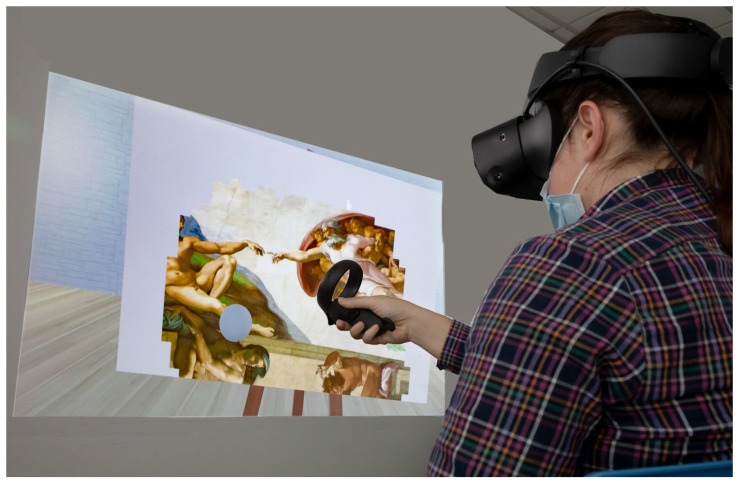
A photo showing a subject using the VR system to unveil the Creation of Adam of Michelangelo. In this photo the therapist could check the performance of the patient, as projected on a wall.

As shown in Figure 1, during the virtual art therapy session, each subject sat wearing an Oculus Rift Head Mounted Display, and held, in his/her paretic hand, an Oculus Controller joystick, which allowed them to interact with the virtual stimuli (by a customized script in C#). The virtual environment, designed by using 3ds MAX 2018 and implemented in Unity 2018 game engine software, consisted of a large and comfortable room, in the middle of which was a canvas (40 cm × 60 cm) on an easel. The subject could interact with the canvas with a spherical brush, displayed in VR in the same place as the participant’s real hand [15]. Each virtual canvas appeared white at the beginning of the task. Patients were instructed that the brush could color the canvas when put in contact with it, forming a painting. The illusion to paint an artwork was given thanks to a white, thin, virtual panel (composed by 247 white pixels) placed in front of the canvas, which occluded the visibility of the underlying paintings. When the subject touched the virtual panel, the target pixels were automatically deleted, allowing them to see a part of the underlaying painting, which was a historical, artistic masterpiece. Patients performed all the different trials that they were able to complete within a session, and each trial was related to a different painting (for example, the Annunciation of Leonardo, the Birth of Venus of Botticelli, the creation of Adam of Michelangelo, the bedroom of van Gogh, and so on). All the selected paintings are associated with a concept of beauty, as verified in previous studies on the aesthetic judgments given by healthy subjects about these paintings [15,23]. The type of task, the size of the canvas and its distance from the subject were planned to allow the patient to perform upper limb movements that were helpful for arm and hand recovery [15]. All the canvases had a rectangular shape (60 cm × 40 cm), and the longer side could be made horizontal or vertical, which mainly required shoulder ab–adduction or flexo-extension, respectively. The choice was performed by the therapist, according to the personalization of the therapeutic aims for each session. Our previous data showed that the time to complete each trial during the first session ranged between 9 and 33 s (depending on the painting), with the virtual brush staying on the canvas for 5–11 m; both duration and length were progressively reduced with the sessions [15]. Each patient was previously instructed to move their upper limb without moving their trunk. Each trial was controlled by a physiotherapist who monitored the movements of the patient, in order to mitigate trunk compensation strategies, and who also monitored the patient’s performance in the virtual environment on a computer’s monitor. For the patients who were not able to perform the upper limb movements in an autonomous manner in the first sessions of therapy, the therapists helped them, by supporting the weight of the limb (this support was reduced and then removed as soon as possible), analogously to what happens in conventional therapy.

### 2.4. Statistical Analysis

Data are reported in terms of means and standard deviations for continuous measures, and as medians and interquartile ranges for ordinal measures. Given the sample size and the non-normal distribution of most of variables (verified by Kolgomorov-Smirnov analysis) non parametric tests have been used. The Wilcoxon test for paired comparisons (pre vs. post) and the Mann–Whitney U-test for unpaired comparisons between groups (VATG vs. CG) were performed. The efficacy of the interventions was computed in terms of effectiveness [24,25], a parameter representing the obtained improvement, expressed as a percentage of the maximum achievable improvement (going from 0% to 100%; set at −100% for worsening).

The PRPS and fatigue scores were averaged among sessions for each patient, and their correlations were tested using the Spearman coefficient (R). The study was designed as an intention-to-treat protocol; therefore, even if some patients did not perform all the 12 sessions for some clinical reasons (for example, fever) they were analyzed in their allocation group.

## 3. Results

At baseline, the two groups were matched in terms of age (VATG: 68 ± 9; CG: 71 ± 13 years old, *p* = 0.553), gender (50% of women in both groups), time from stroke (VATG: 23 ± 7 days; CG: 23 ± 6 days, *p* = 0.970), type of stroke (90% ischemic stroke and 10% hemorrhagic stroke in both groups; these percentages roughly reflect the incidence proportion of ischemic vs. hemorrhagic stroke reported in the literature: 80% vs. 20% [26]), and the side affected by the stroke (30% of patients had an affected left hemisphere in both groups). The stroke severity was similar between the two groups at baseline (pre-treatment), as shown by the median values of the BI-scores (29 for VATG vs. 28 for CG) and also by the other clinical scores, which were not significantly different between the two groups, as reported in Table 1, which also reports the pre-post within-group comparisons.

**Figure 2:**
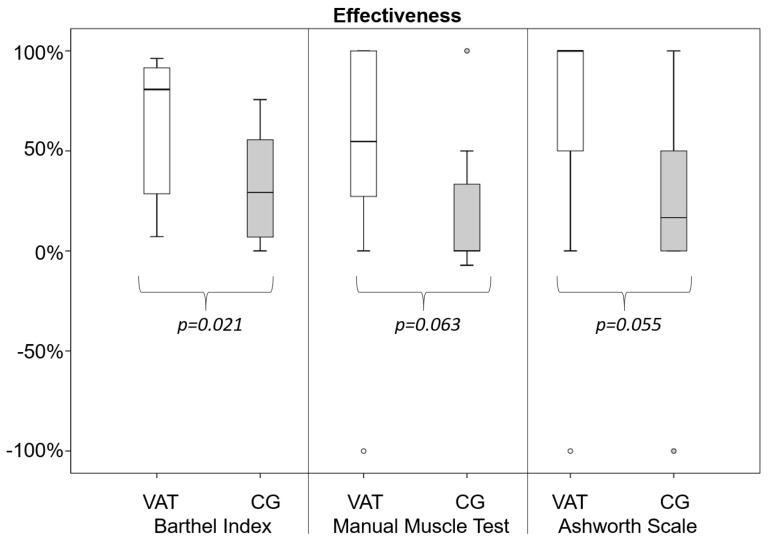
Box–whiskers plot of treatment effectiveness in terms of the virtual art therapy group (VATG, empty boxes) and control group (CG, grey boxes). The boxes represent the 50% of patients within first and third quartiles, with the bold horizontal lines representing the median values. The whiskers show 1.5 times the interquartile range, the dots represent patients out of the whisker ranges. The *p*-values show the results of Mann-Whitney U-tests.

For the experimental group, the mean number of VAT sessions was 10 ± 2 (with a duration of 27 ± 8 days). Figure 1 shows the percentage improvements, in terms of effectiveness, for the two groups and their relevant comparison. A significantly higher effectiveness in terms of BI-score was found for VATG than for CG (Figure 2). For the Manual Muscle test, the effectiveness was not significantly different, but analyzing the effectiveness for MMT-subscores, it was significantly higher in VATG than in CG in terms of pinching movements (66 ± 39% vs. 18 ± 33%, *p* = 0.008). In terms of spasticity, two patients, one in each group, showed a slight worsening of spasticity after treatments. Without these patients, the effectiveness was superior in VATG than in CG (83 ± 35% vs. 35 ± 41%, *p* = 0.031).

Given clinical problems (such as fever), not all the subjects performed the 12 planned sessions of VAT (and not all the subjects in the CG group performed the planned sessions of conventional therapy). According to an intention-to-treat protocol, this did not change the statistical approach, but it should be reported that the mean number of performed VAT sessions was 10 ± 2. Participation in virtual art therapy showed very high rates among patients (mean PRPS-score: 5.7 ± 0.3, with a maximum of 6) and it was negatively and significantly correlated with the perceived fatigue (R = −0.794, *p* = 0.006,on average 2.6 ± 2.0, with a maximum of 10). 

## 4. Discussion

In the present study, we aimed to test the efficacy of the virtual art therapy (VAT) protocol, based on neuroaesthetic principles and the Michelangelo effect, in improving the autonomy of patients with stroke in their activities of daily living and upper limb functioning. The adopted pilot protocol included 12 sessions (3 per week) of virtual art therapy. The obtained findings showed that the group of patients treated for one month with the virtual art therapy improved their level of upper limb muscle strength, in particular, in terms of pinching, as well as increased their independency in activities of daily living. The good outcomes in terms of grip strength of the hand can be explained by the trained functions necessary to keep the virtual brush in one’s hand. The control group treated with conventional therapy did not show a significant improvement in upper limb muscle strength, but also, for these patients, the improvement in terms of independency was statistically significant. The BI-score was significantly higher for VATG than for CG. Regarding spasticity, the difference between the two groups was not statistically significant, due to two patients, one per group, who had a slight worsening that increased data variability. For the other patients who improved in terms of spasticity, a higher effectiveness was observed for VATG. However, it should be noted that, despite not being statistically significant, spasticity was quite lower at baseline in the VATG group than in the CG group. Further studies should mitigate this potential bias, by enrolling more subjects to obtain two more homogenous groups to compare. It should be noted that our VAT protocol can be used also in the presence of slight or moderate hand spasticity, because it does not hamper the grasping of the joystick. Two reviews regarding spasticity post stroke reported a prevalence in the first month ranging between 4 and 46% of patients, with severe spasticity occurring only in 2–2.6%, but these percentages progressively increased in the late acute phase and chronic phase of stroke [27,28].

These results are in line with the previous preliminary results suggesting the potentially rehabilitative effects of a protocol of virtual art therapy, based on the Michelangelo effect [1]. This effect allowed patients to perceive less fatigue during rehabilitation and to perform more accurate movements [15]; pinching movements were the most improved type of action. A previous study on video-game-based therapy for children showed an opposite result: fine motor functions improved more with conventional therapy than with video-game based therapy, which improved gross motor functions more [29]. However, differently from our present study, in that one, the subjects interacted with the video games through total body movements, and fine hand movements were not tracked by the computerized system. If, for children, gaming could be the keystone to improve their engagement and their active participation in the therapy sessions [30], for older people, art could play a similar role. Participation (defined as the active participation in the proposed exercises with maximal effort, as well as taking interest in them and future therapy sessions [21,22]) is fundamental for obtaining a good outcome in neurorehabilitation [31]. Previous findings showed that this VAT protocol reduced the perceived fatigue in patients with stroke [15]; here, we found that a lower fatigue was significantly correlated with a higher participation in the therapy sessions, and that the adherence to the protocol allowed the participants to obtained better outcomes. Participation [15,21], fatigue [32], and general psychological factors [33] are fundamental aspects to take into account when a new technological approach has been introduced to neuro-rehabilitation.

The improvements obtained with the proposed virtual art therapy protocol could conceivably be explained, in light of the recent neuroaesthetics literature. First of all, they could be explained by the Michelangelo effect; the interaction with art reduces the fatigue perceived by the patient, and improves their performance of fine movements [15]. Then, the activations of the motor and pre-motor networks, as observed in people observing artworks [5,6,7,8], could act as enhancers of the activations needed by the patients to unveil the paintings in our protocol. Additionally, it has been found that art-based therapies and interventions can effectively be used to reduce pain, stress, depression, breathlessness and other symptoms in patients with several medical conditions [31]. Last, but not least, despite us not assessing cognitive functions, the scientific literature reports the possible use of art therapy to improve these domains [34], also reporting that some diseases may improve the personal disposition of neurological patients to produce art [35]. Further studies are needed to deeply investigate these aspects that were not evaluated in our study. In particular, it will be interesting to investigate what would happen in a brain, damaged by a stroke, during the contemplation of an artistic masterpiece. Our hypothesis that is needed to be confirmed, is whether art could stimulate the motor and premotor networks in a damaged brain as well, promoting neuroplasticity.

### 4.1. Limitations and Considerations

There are some limitations that need to be taken into account in our study. The most important limitations, strictly intertwined with each other, included the fact that this study was not a registered randomized controlled trial, as well as the reduced sample size. Even though the appropriate number of enrolled patients was computed according to a power analysis, based on previous data [23], and was in line with other previous trials [10,29], it was too small to design a randomized controlled trial. So, we preferred a matched case–control design. In the future, a pre-registered randomized controlled trial should be conducted, and this could also improve the limited statistical significance of some results, for example, with respect to the differences in the effectiveness for the MMT total score and Ashworth-score that, in the presented study, only approached the statistically significant threshold.

Second, in order to avoid additional cognitive load, mental fatigue and a prolonged duration of the whole protocol, we did not include specific questionnaires for measuring the perceived level of sense of presence; instead we treated this factor as a default state elicited by immersive virtual reality. Thus, future studies are need to better understand the relationship between the sense of presence and the effectiveness of the rehabilitation treatment.

Additionally, it is important to note that our participants could naturalistically interact with the virtual canvas by using the dedicated VR controller, but, in the virtual environment, the participant would just see a virtual spherical brush, not the virtual reproduction of his/her real head and real upper limb. Recent evidence suggests that the substitution of the real body with a virtual one is able to elicit the sense of embodiment, i.e., the illusory feeling that the virtual body is one’s “own” body [36,37]. Recent findings also suggest that the type of the embodied virtual body could affect the perception of one’s own abilities [14].

With the present version of the virtual art therapy protocol, we did not include a virtual body, and thus we could not investigate as to whether the sense of embodiment could affect and boost the effectiveness of the treatment.

Finally, we did not collect some possibly important clinical information, such as the number of patients who received thrombolysis or thrombectomy in acute units before being transferred in our rehabilitation unit, in which they started the therapies. However, it should be considered that patients needing intensive therapies after having received thrombolysis or thrombectomy are those for whom these interventions were not or only partially effective in reducing the sequelae of stroke, as confirmed by the wide body of literature reporting these interventions as not statistically significant in predicting the outcome of patients needing intensive rehabilitation [38]. Hence, this study should be considered as a preliminary experience that can be used to plan a registered, randomized controlled trial with a follow-up assessment, in order to verify the maintenance of the obtained benefits (using our data, setting an alpha level at 5% and power of test at 80%, a calculated sample size of 26 patients was found to be necessary to obtain statistically significant differences, also in terms of Barthel Index effectiveness). This computed sample size (26 patients) was quite larger than that of the present study (19 patients), estimated on the basis of previous results [15]. This difference is conceivably due to the fact that the sample size of the present study was computed using the root mean square error of kinematic movements as a predictor of functional outcomes (BI-score and MMT-score). Preliminary data on the BI-score and MMT-score were not available, so that choice was a forced approximation that provided an estimation (*n* = 19), which was not too far off from the one re-computed in light of our results (*n* = 26), and it is probably a more accurate estimation of the needed sample for a further randomized controlled trial.

### 4.2. Implications of Using Virtual Reality Technology in Clinical Practice

The use of virtual reality technology in clinical practice requires some attention that we would briefly discuss. First, regarding some technical aspects, the clinical team needs a dedicated space for placing the instruments, which include the VR-dedicated PC, cables, the VR headset and the physical space where the patients can perform the exercises. Therefore,, we needed to plan and optimize the physical space for a better use of VR technology. We performed all the sessions in a dedicated room, so that the patient was not in a noisy gym. Consequently, we also suggest dedicating time to instruct the clinical team in using this technology, to learn any potential aversive effect that the system could induce (e.g., cyber sickness), and to prevent possible compensation strategies. The time of exposure in the virtual environment is another point to discuss. Indeed, considering the fragility of the patients and their poor experience with new technology, we planned sessions of about 30 min (plus the preparation), in order to avoid any dissociative state, cyber sickness or any other aversive effect. Finally, the most important aspect for us to consider was to identify the neuroscientific principles to take into account when the VR scenario was adopted. In our case, the VR was the method we used to apply neuroaesthetic principles to therapy: for this reason, we referred to the efficacy of art therapy administered by means of VR, and not the efficacy of VR per sè.

## 5. Conclusions

Art therapy has a long story of successes [1], but their neuroscientific explanations are not clear yet, and an emerging discipline such as neuroaesthetics could be helpful to clarify the effects of art on damaged brains. Virtual reality could be a valid tool to bring art from museums to hospitals, immersing patients in artistic environments and giving them the illusion that they are observing or even creating an artistic masterpiece.

From a clinical point of view, our study showed that a protocol of virtual art therapy lasting 1 month may enhance the recovery of independence by the patient, with stroke in the subacute phase. The alliance of art therapy, neuroaesthetics and new technologies (also including artificial intelligence [38,39,40]) may favor the development of new, more effective, protocols of neurorehabilitation.

## Figures and Tables

**Table 1:** Medians and interquartile ranges (third quartile minus first quartile, reported between brackets) of pre- and post-rehabilitation clinical scale scores (MMT stands for Manual Muscle Test) in the virtual art therapy group (VATG) and control group (CG). Pre–post comparisons for each group were performed with the Wilcoxon test, and the relevant within-group *p*-values statistically significant if <0.05.

Clinical Scale Scores	VATG			CG		
	Pre	Post	*p*-Value	Pre	Post	*p*-Value
Ashworth scale	1 (1)	0 (1)	0.187	2 (2)	1 (1)	0.180
MMT-shoulder	3 (2)	4 (1)	0.067	3 (3)	3 (2)	0.317
MMT-elbow	3 (1)	4 (2)	0.075	4 (3)	4 (2)	0.102
MMT-pinch	3 (3)	5 (2)	0.006	4 (3)	4 (3)	0.083
MMT-total	10 (5)	13 (5)	0.020	10 (9)	11 (7)	0.141
Barthel Index	29 (18)	88 (16)	0.005	28 (38)	72 (31)	0.012

## Data Availability

Anonymous data are available on request to send to the corresponding author.
